# The γ-TuRC: mastermind of multipurpose actions at the centrosome

**DOI:** 10.1007/s00018-026-06080-w

**Published:** 2026-02-19

**Authors:** Swarnendu Mukhopadhyay, Qi Gao, Tapas K. Manna, Elmar Schiebel

**Affiliations:** 1 School of Biology, Indian Institute of Science Education and Research, Vithura, Thiruvananthapuram, Kerala 695551 India; 2 Zentrum Für Molekulare Biologie der Universität Heidelberg (ZMBH), Deutsches Krebsforschungszentrum (DKFZ)-ZMBH Allianz, Universität Heidelberg, Heidelberg, 69120 Germany; 3 Heidelberg Biosciences International Graduate School (HBIGS), Universität Heidelberg, Heidelberg, 69120 Germany

**Keywords:** Centrosomes, Centriole duplication, γ-tubulin complex proteins, SAS-6

## Abstract

The γ-Tubulin Ring Complex (γ-TuRC) comprised of γ-tubulin and γ-tubulin complex proteins (GCP2-6) serves as the main protein scaffold for microtubule nucleation in most animal cells. In lower eukaryotes such as *Saccharomyces cerevisiae*, a simpler scaffold consisting of γ-tubulin and GCP2-3 bestows its microtubule nucleating function. Although most microtubules in interphase cells are nucleated at the centrosome, this process relies on only a small fraction of the total soluble γ-tubulin that becomes recruited and activated there. A number of accessory proteins have been identified to be involved in the recruitment, stabilization and activation of the γ-TuRC presumably at the right time and subcellular region of the centrosome. Though, function of the γ-TuRC was attributed to microtubule nucleation by the pericentriolar material of the centrosome, very recent studies have uncovered its localization inside centrioles. Within centrioles, γ-TuRC interacts with the eight-subunit augmin complex. Its localization to the inner centriole suggests that γ-TuRC and augmin possess functions beyond microtubule nucleation. Moreover, the levels and organization of γ-TuRC differ markedly between fully mature and growing centrioles, indicating cell cycle–dependent recruitment and functional reprogramming during centriole biogenesis. Finally, we discuss how mutations in γ-TuRC genes impact development and are linked to cancer progression.

## Introduction

### The γ-TuRC and microtubule nucleation

Microtubules, together with actin and intermediate filaments, constitute one of the three main components of the eukaryotic cytoskeleton. They fulfill a wide range of essential functions, including cellular organization, intracellular transport, cell migration, and chromosome segregation during cell division. Structurally, microtubules are composed of linear chains of α/β-tubulin heterodimers arranged into protofilaments. In most organisms, 13 tubulin protofilaments assemble into a hollow cylindrical structure, with a dynamic plus end terminating in β-tubulin and a more stable minus end capped by α-tubulin.

Microtubules can assemble de novo from α/β-tubulin subunits through a process known as microtubule nucleation. γ-tubulin, a member of the tubulin superfamily, was first identified in *Aspergillus nidulans* as a suppressor of a β-tubulin mutant by Oakley and colleagues, and was soon recognized as a universal microtubule nucleator in eukaryotes [1, 2]. The molecular mechanism of γ-tubulin function became clearer after structural analysis of the in vitro oligomerized budding yeast γ-tubulin small complex (γ-TuSC). The tetrameric yeast γ-TuSC is composed of two γ-tubulin complex proteins, GCP2 and GCP3, that have two conserved domains. The N-terminal GRIP1 domain enables interactions between GCPs, while the C-terminal GRIP2 domain binds to γ-tubulin. In vitro, the yeast γ-TuSC can assemble into spirals that match microtubules in both pitch and diameter [3]. Based on this structural analysis and the observed interaction between γ-tubulin and α-tubulin at the microtubule minus end, Agard and colleagues proposed the now widely accepted “template model”, in which 13 γ-tubulin molecules in the oligomerized γ-TuSC interact directly with α/β-tubulin to seed formation of a microtubule with 13 tubulin protofilaments [4]. Later it was found that in vivo 7 γ-TuSC molecules oligomerize at the spindle pole body (SPB), the yeast microtubule-organizing center (MTOC), upon binding to the SPB protein Spc110 into a γ-tubulin ring complex (γ-TuRC) with 13 exposed γ-tubulin molecules [5–8]. Spc110 carries a conserved CM1 motif (centrosomin motif 1) that is able to crosslink γ-TuSC molecules within the γ-TuRC oligomer [9].

**Fig. 1:**
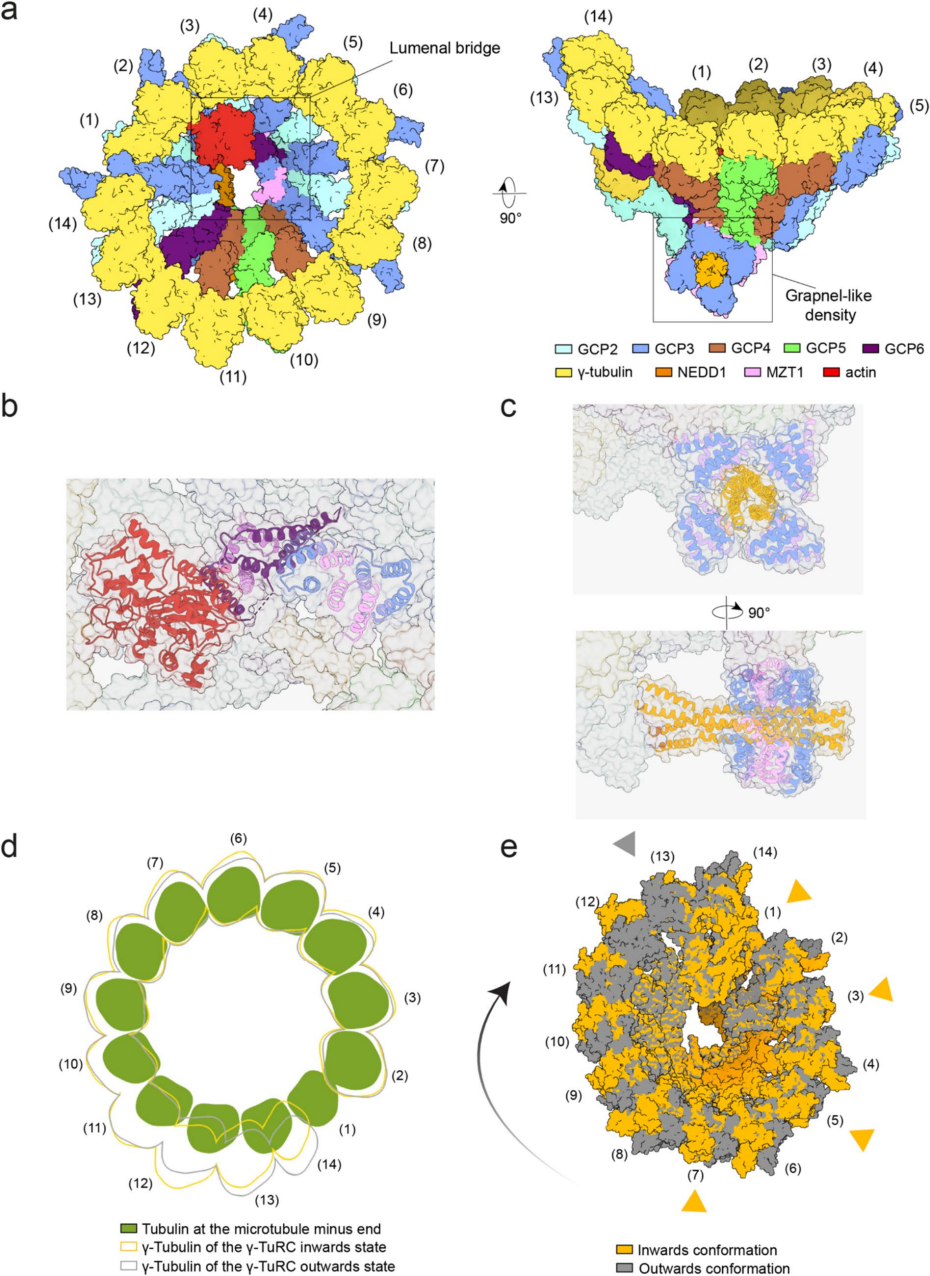
Structure and conformation of vertebrate γ-TuRC. **a** Schematic presentation of the cryo-EM structure of the *Xenopus laevis* γ-TuRC shown in two views. Coloring as indicated. Spoke numbers are labeled. Molecular representations were generated from PDB 9I8N and EMD-52730. The lumenal bridge and grapnel-like density are highlighted. **b** Structure of the lumenal bridge, consisting of two MZT1 modules (pink), the N-terminus of GCP3 from spoke 8 (blue), the N-terminus of GCP6 (purple), and one molecule of actin (red). **c** Structure of the grapnel-like density, composed of four copies of the C-terminus of NEDD1 (orange), the N-terminus of GCP3 (blue), and MZT1 (pink). **d** Comparison of the arrangement of αβ-tubulin subunits within a microtubule (green) with the γ-tubulin molecules of the γ-TuRC in their inward (orange) and outward (gray) states. The diagram illustrates that the vertebrate γ-TuRC deviates from perfect microtubule symmetry. **e** Overlay of the inward (orange) and outward (gray) γ-TuRC conformations. Orange triangles mark the GCP2-containing spokes that interact with the CM1 modules of CDK5RAP2 (CDK5 Regulatory Subunit–Associated Protein 2) in the inward state. In contrast, a γ-TuRC bound by CDK5RAP2 solely at GCP2 in position 13 adopts the outward conformation. The black arrow indicates the movement from outward to inward state. Molecular representations were generated from PDB 9I8G and EMD-52718 (inward conformation) and PDB 9I8H and EMD-52719 (outward conformation)

The *Xenopus laevis* γ-TuRC, first isolated by Zheng and Mitchison in 1995 [10], was found to be more complex than its yeast counterpart, containing five distinct GCP paralogues (GCP2–GCP6), all of which bind γ-tubulin via their GRIP2 domains [11]. However, despite extensive research over the following 25 years, detailed structural data remained elusive in particularly about the order of the GCPs within the γ-TuRC. In 2020, several groups successfully described the structure of isolated γ-TuRCs from *Xenopus laevis* egg extract and human cells (Fig. 1a) [12–15]. These studies consistently revealed a conserved GCP organization: (GCP2-GCP3)₄–GCP4–GCP5–GCP4–GCP6–(GCP2-GCP3)₁, with each GCP bound to a γ-tubulin molecule.

These structural studies also showed that the extended N-terminal domain of GCP6 bound to the microprotein MZT1 (mitotic-spindle organizing protein associated with a ring of gamma-tubulin 1), a protein of only 82 amino acids in humans. In combination with the N-terminus of GCP3 at spoke 8, the GCP6-MZT1 and GCP3-MZT1 interactions form the lumenal bridge, which spans the interior of the γ-TuRC from spoke 12, where the core of GCP6 is localized, to spoke 2 (Fig. 1a and b) [12–15]. In addition to GCPs and γ-tubulin, the vertebrate γ-TuRC contains one molecule of actin bound to position 2 via the MZT1-N-GCP6 module [12–15] (Fig. 1a and b). MZT1-N-GCP3 modules are also important for the binding of the adaptor protein NEDD1 (Neural precursor cell expressed, developmentally down-regulated 1) to the γ-TuRC (Fig. 1c). Notably, unlike the oligomerized yeast γ-TuSC, the vertebrate γ-TuRC deviates from perfect microtubule symmetry (Fig. 1d). This deviation is most pronounced at positions 9–12, which include the GCP4–GCP5–GCP4–GCP6 core [12–15]. This structural asymmetry raises the question of how the vertebrate γ-TuRC functions as a template for microtubule formation. As discussed below, binding of γ-TuRC activators such as CDK5RAP2 may affect the structure of the γ-TuRC (Fig. 1e).

**Fig. 2:**
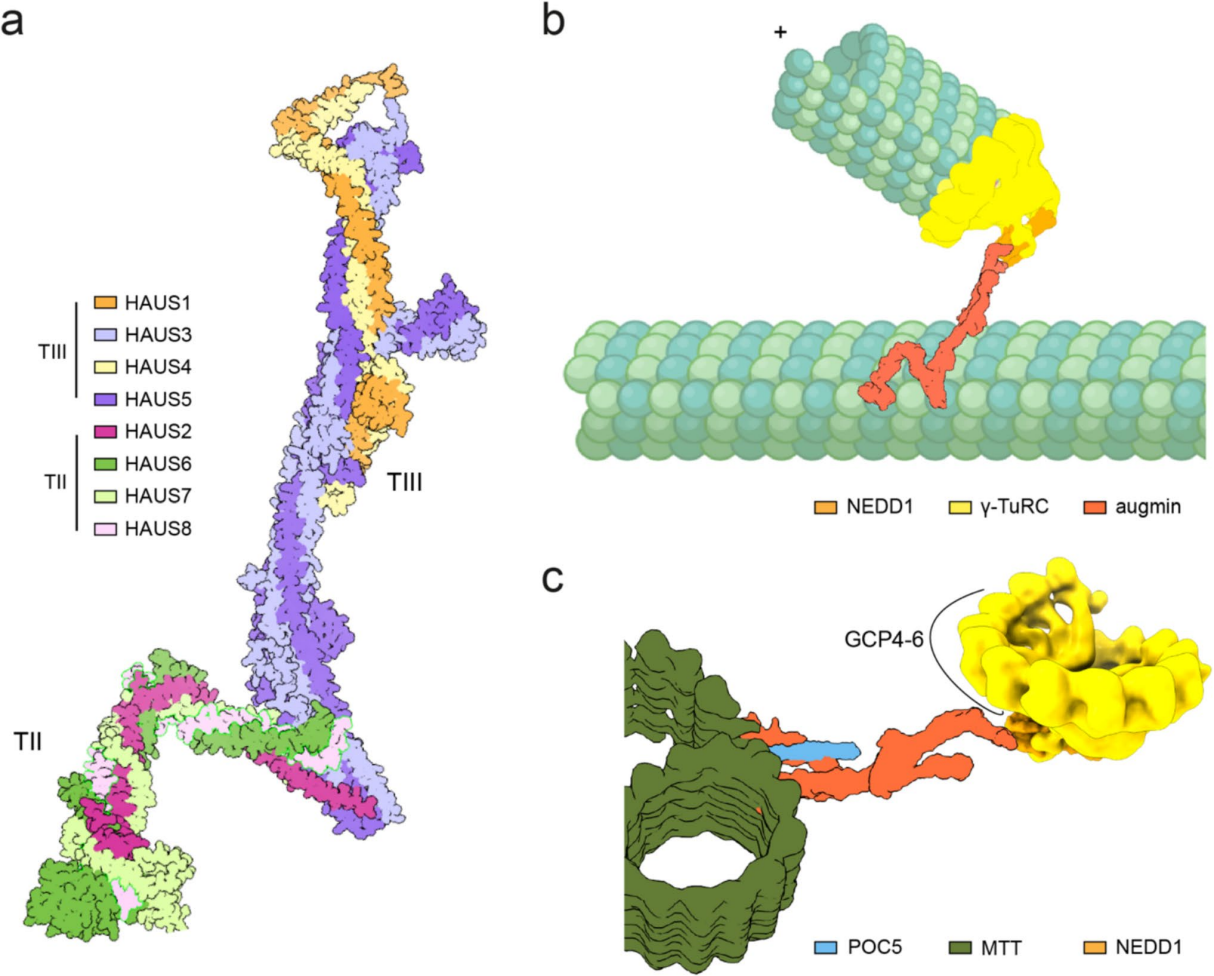
Structure and function of augmin complex. **a** Cryo-EM structure of the augmin complex showing the TII (HAUS2, HAUS6, HAUS7, and HAUS8) and TIII (HAUS1, HAUS3, HAUS4, HAUS5) subunits. Colors are indicated. **b** Schematic model of the augmin-mediated microtubule branching process. The TII of augmin anchors the complex to a pre-existing mother microtubule, providing the structural platform for branching. The TIII then binds to γ-TuRC through NEDD1, positioning the γ-TuRC to initiate nucleation of a branched daughter microtubule. Colors are indicated. **c** Schematic model illustrating the organization of the POC5-augmin-γ-TuRC complex within the centriolar lumen. Recruitment of the luminal augmin-NEDD1-γ-TuRC complex depends on the inner scaffold protein POC5. The augmin TII directly interacts with POC5 and is positioned closer to the microtubule triplet wall. In contrast, the augmin TIII engages γ-TuRC through the NEDD1/N-GCP3/MZT1 grapnel-like structure, orienting the complex toward the centriolar lumen. The polarity of the γ-TuRC within the centriolar lumen is indicated. Colors are indicated

The eight-subunit augmin complex (HAUS1–8) cooperates with the γ-TuRC and is essential for branched microtubule nucleation, mitotic spindle assembly, and the anchoring of the centriole lumen [16–18] (Fig. 2a, b and c). The augmin subunits were first identified by Goshima et al. in a screen for factors required for bipolar spindle formation [19, 20]. Subsequent studies revealed that during mitosis, augmin binds directly to spindle microtubules and recruits the γ-TuRC through its interaction with the γ-TuRC recruitment factor NEDD1, thereby promoting the formation of branched microtubules at defined angles relative to the parent microtubule [21–23] (Fig. 2b). Recent investigations employing ultra-expansion microscopy and cryo-electron tomography have uncovered an unanticipated role of augmin within the centriole lumen, while also resolving the structure of the augmin complex and its association with the γ-TuRC [24–28].

### The human centrosome

**Fig. 3:**
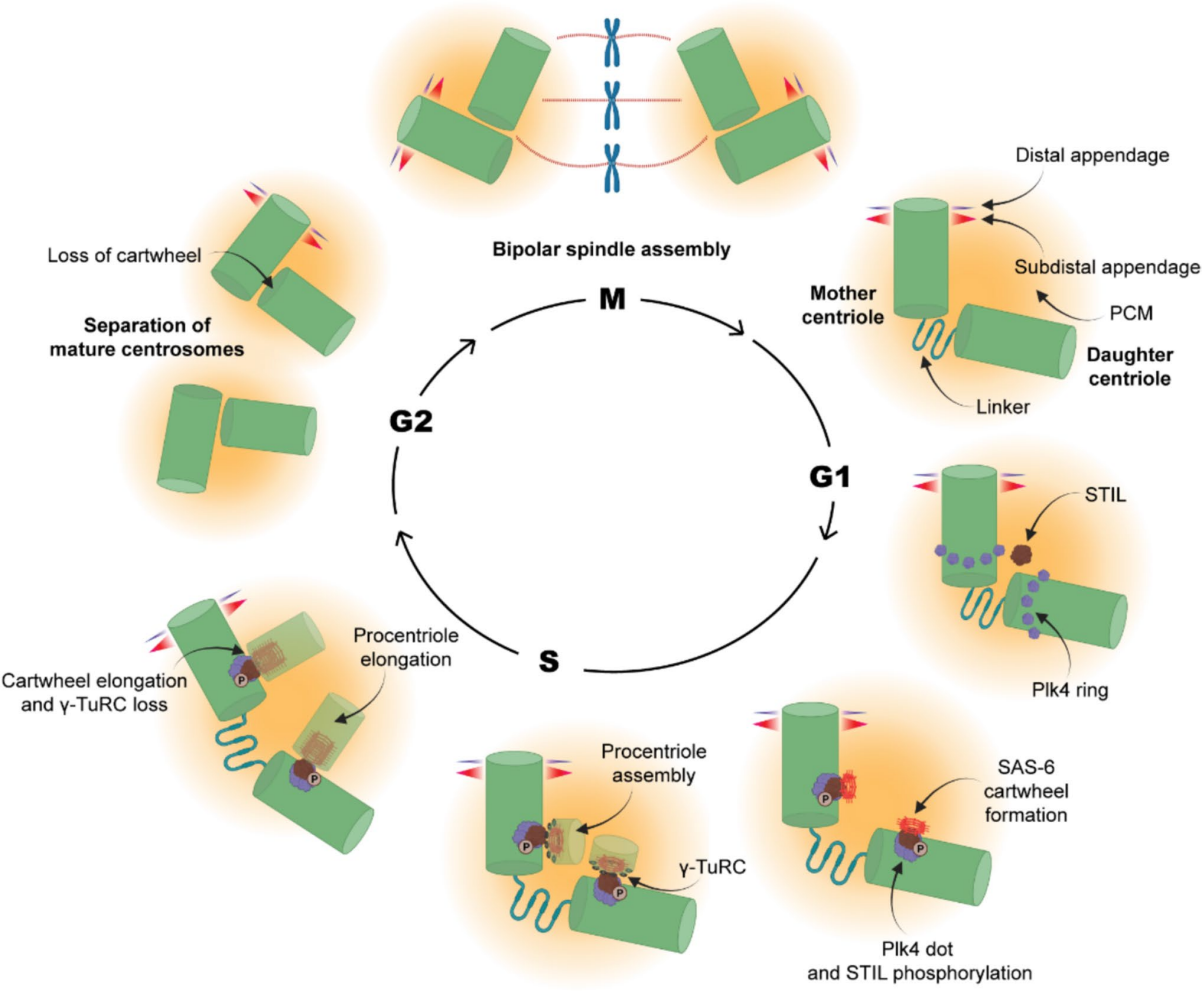
The centriole duplication cycle. The mother centriole typically consisting of its appendages and the daughter centriole are connected with the centrosome linker and embedded in the PCM at early G1. At the onset of G1/S transition, Plk4 kinase initially gets localized as diffused ring around proximal region of each parental centrioles and thereafter, gets concentrated at a single site, where STIL is recruited. Plk4-mediated STIL phosphorylation activates its binding to SAS-6 and formation of the cartwheel of nine-fold symmetry. γ-TuRC recruitment to the cartwheel presumably activates microtubule nucleation of the newly emerged centriole (pro-centriole). The pro-centriole further elongates until it gets matured at G2. The linker between the parent centrioles is lost at this stage and separation of the duplicated centrosomes begins. During M-phase, the duplicated centrosomes migrate to two opposite poles that constitute the bipolar mitotic spindle

Proper microtubule assembly and organization are essential for fundamental cellular processes such as cell division, cell polarization, and cell motility. Microtubule-organizing centers (MTOCs) [29] that harbor the γ-TuRC spatially regulate microtubule assembly. In most animal cells, the centrosome serves as the primary MTOC and it is constituted by a stable microtubule-based cylindrical structure called the centriole, embedded in a cloud of pericentriolar material (PCM) proteins [30, 31]. At the beginning of S phase, the two centrosomes undergo a duplication cycle, with each parental centriole initiating the formation of a new daughter centriole from its proximal side. By recruiting specific proteins, the new centriole matures from the S phase through the end of mitosis, developing into a fully functional centrosome capable of microtubule nucleation and centriole duplication [32]. During mitosis, each mother centriole, together with its attached daughter centriole, forms a spindle pole of the mitotic spindle (Fig. 3). At the end of mitosis, the newly formed centriole detaches from the mother. The frequency of centrosome duplication has to happen strictly once during each cell cycle in order to organize bipolar microtubule spindles and faithful segregation of the genetic contents [33, 34].

The centrioles display a characteristic nine-fold symmetry composed of triplet microtubules—designated A, B, and C. This distinctive arrangement is established through the assembly of these triplets around a nine-fold symmetric, cartwheel-like protein scaffold that originates from the proximal side of each parental centriole [31, 35, 36]. The cartwheel, primarily formed by the oligomeric assembly of nine dimers of the SAS-6 protein (spindle assembly abnormal protein 6), is initiated by the recruitment of SAS-6 to the nascent centriole (procentriole) assembly site on the surface of the parental centriole, a process activated by Polo-like kinase 4 (Plk4) [37–39] (Fig. 3). Plk4 initially gets localized at the proximal end of the mother centriole by encircling it as a diffused ring. Subsequently, the ring gets transformed into a concentrated dot-like structure, which recruits and phosphorylates the protein STIL (SCL interrupting locus) to the procentriole assembly site. Plk4 phosphorylated STIL, specifically in its C-terminal STAN (signal transduction and activation of transcription) domain, facilitates the recruitment of its binding partner SAS-6, which oligomerizes into the cartwheel-like arrangement with its further growth as multiple stacks [40–43]. Oligomerization of the N-terminal globular head domains and the following coiled-coil regions of nine SAS-6 dimers gives rise to the cartwheel-like framework from where the procentriole begins to assemble (Fig. 3) [44]. Additional proteins such as CEP135, interact with the periphery of the cartwheel and form the pin head that interacts with centriole microtubules [45].

**Fig. 4:**
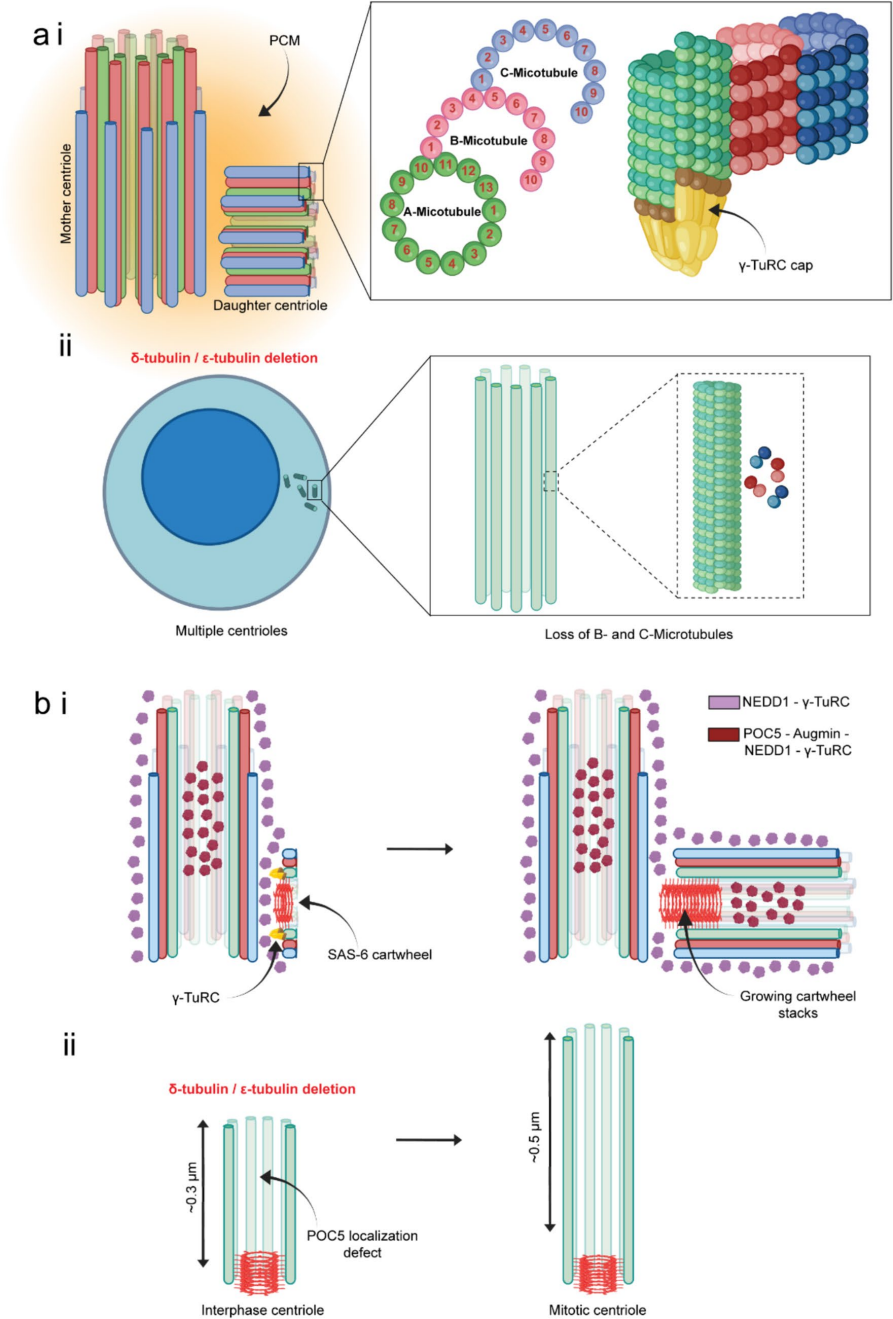
Assembly and organization of centriolar microtubules and other centriole biogenesis proteins. **a i** Assembly and organization of A, B and C microtubules of centrioles. The A-microtubule consists of 13 protofilaments, whereas B- and C- microtubules have 10 protofilaments each. The side view of the microtubules shows presence of a γ-TuRC like cap at the minus end of the A- microtubule. **a ii** Multi-centrioles defect in the cell deleted of δ- or ε-tubulin. The centrioles in the δ- or ε-tubulin-deleted cells possess only singlet microtubules instead of triplets. **b i** Dynamic organization of centriolar proteins, SAS-6, NEDD1, Augmin, POC5 and γ-TuRC during the assembly of new centriole from the matured mother centriole. Early stage of procentriole formation shows NEDD1 and γ-TuRC localized in the PCM and the outer wall of both the mature centriole and the nascent centriole (procentriole), whereas each of POC5, Augmin, NEDD1 and γ-TuRC also appear to localize inside the lumen of the mature centriole. As the growing centriole reaches an intermediate length, the SAS-6 cartwheel appears to grow further in both directions and the POC5-Augmin-NEDD1-γ-TuRC axis is seen to localize in the growing centriole lumen. **b ii** Loss of luminal POC5-Augmin-NEDD1-γ-TuRC axis in the δ- or ε-tubulin-deleted cells possessing multi-centrioles with singlet microtubules in each. These centrioles with singlet microtubules appear to grow further during progression from interphase to mitosis and SAS-6 seems to remain associated

Procentriole assembly involves the nucleation and elongation of nine sets of triplet microtubules from the outer edges of the SAS-6 cartwheel stacks, and such unique mode of organization gives rise to the stable architecture of the centriole. Unlike the symmetric organization of 13 protofilaments of normal microtubules, centriolar triplet microtubules, however only exhibit a partially symmetric nature of protofilament organization (Fig. 4). While the A-microtubule, which is located most interior side of the centriole cylinder, organizes with 13 protofilaments, the B-microtubule, which appears juxtaposed to A-microtubule and the C-microtubule located closely with the B-microtubule, consists of 10 protofilaments each [46]. In addition, a number of microtubule inner proteins (MIPs) localize inside the A, B and C centriole microtubules at defined positions, contributing to their stability and function [47].

Though the molecular mechanisms that favour such uniquely asymmetric protofilament organization is just emerging as discussed below, the specific protein linkers connecting the A- and C-microtubules provide the integrity of the triplet microtubule organization in the proximal region of the new centriole [48]. Recent studies have identified several proteins likely involved in forming the A–C linker, including CCDC77, WDR67, and MIIP [49]. Knocking down these proteins individually and in combination, resulted in the destabilization of a significant portion of the proximal part of the centrioles, supporting their role in maintaining the structural stability of the proximal microtubule wall. Furthermore, the localization of these proteins at the A-C linker has been shown to be mutually dependent, as the loss of one protein leads to the removal of the others. Additionally, the inner scaffold protein POC5 [50], also contributes to the stability of centrioles, as its loss partially destabilizes the microtubule wall and demolishes the centriole completely when they are co-depleted with the A-C linker proteins [49]. It is also interesting to note that depletion of A-C linker proteins causes destabilization of the torus structure around the procentriole wall constituted by CEP152 and CEP63, and associated abrogation of the cartwheel initiating proteins Plk4 and STIL, and the cartwheel protein SAS-6 [49]. However, the direct molecular connections between the A-C linkers and the assembly of the procentriole cartwheel remain to be investigated in the future.

### Recent developments

#### The γ-TuRC at the pericentriolar material of centrioles

In vertebrate cells, centrosomes act as the primary MTOCs by recruiting and probably activating γ-TuRCs. The universal function of the γ-TuRC at the MTOC is the de novo formation of microtubules from α/β-tubulin subunits. In addition, the γ-TuRC determines the number of protofilaments, establishes microtubule polarity within cells, and regulates microtubule formation in space and time. As such, all γ-TuRC core genes are essential for the viability of human cells [16].

In light of these essential functions at centrosomes, it is surprising that the majority of γ-tubulin is not associated with centrosomes [51, 52]. Cell fractionation studies of asynchronized cultures estimate that ~ 80% of γ-tubulin resides in the cytoplasm, with only ~ 20% localized at centrosomes [52]. Even more strikingly, GFP-based intensity measurements in interphase cells suggest that the centrosomal pool comprises merely ~ 1% of total cellular γ-tubulin [51]. Both approaches, however, have limitations: fractionation depends on immunoblot quantification, which may underestimate centrosomal γ-tubulin due to sample loss, incomplete recovery, or non-linear detection, GFP-based analysis was conducted in a knock-in cell line carrying one copy each of wild-type γ-tubulin and γ-tubulin–GFP. This approach assumes that the fusion protein is fully functional and faithfully mirrors endogenous γ-tubulin behavior. However, this assumption has been challenged for other GFP-tagged fusion proteins [53]. Determining the precise spatial distribution of γ-TuRCs and their assembly intermediates throughout the cell cycle is a critical goal for future research.

The γ-TuRC is a soluble, cytoplasmic complex, raising the question of how it is recruited to centrosomes and other cellular structures such as spindle microtubules. NEDD1, also known as GCP-WD, is a key γ-TuRC–targeting factor whose depletion results in the complete loss of the γ-TuRC from centrosomes and spindle microtubules indicating its essential targeting role [54]. To achieve this targeting function, NEDD1 interacts on the one hand with the γ-TuRC and on the other hand with CEP192 at the PCM, and inside centrioles and along microtubules with the augmin complex [25, 26, 55]. Consistent with this function, CEP192 depletion results in the nearly complete loss of the γ-TuRCs and NEDD1 from the PCM and strongly impairs microtubule nucleation activity of centrosomes [56]. The interaction between NEDD1 and CEP192 is likely mediated by the WD40 domain of NEDD1 and a binding site within the N-terminus of CEP192 located at the core of the PCM, although structural data confirming this interaction are still lacking [57]. In addition to NEDD1, the N-terminus of CEP192 interacts with other proteins, including PLK1, Aurora A, and PLK4 kinases [58]. Whether these kinases phosphorylate NEDD1 or γ-TuRC subunits to regulate microtubule nucleation remains unknown. Another likely binding partner of NEDD1 is the cartwheel protein SAS-6, which plays a key role in centriole duplication (see below) [59].

A key question is how NEDD1 interacts with the γ-TuRC. Previous work suggested an interaction between the C-terminus of NEDD1 and the MZT1–N–GCP3 modules of the γ-TuRC, but the structural arrangement of this interaction remained unclear [23, 60]. Two recent studies have resolved the structure of the NEDD1 in complex with the γ-TuRC, providing important insights into how NEDD1 interacts with the γ-TuRC (Fig. 1c) [25, 61]. Importantly, these analyses were conducted using cryo-electron microscopy of recombinant human and purified *Xenopus laevis* γ-TuRCs [25, 61] in combination with cryo-electron tomography of γ-TuRCs bound to purified centrosomes [25]. In all cases, the underlying binding mechanism between γ-TuRC and NEDD1 was the same indicating that purification or co-expression did not affect the γ-TuRC-NEDD1 interaction. Cryo-electron microscopy revealed that the C-terminus of NEDD1 forms a stable, tetrameric alpha-helical bundle that associates with four MZT1–N–GCP3 modules derived from GCP3 molecules at positions 2, 4, 6, and 14, forming a grapnel-like structure at the base of the cone-shaped γ-TuRC. Within the grapnel, MZT1-N–GCP3 engages the C-terminus of NEDD1 through electrostatic interactions (Fig. 1c). The grapnel itself interacts with the bottom part of the γ-TuRC containing the GRIP1 domains of the GCPs. The two proximal MZT1-N-GCP3 modules dock to specific extensions of GCP5 and GCP6 just N-terminal to the GRIP1 domain [25, 61]. In this instance, it is MZT1 and N-GCP3 of the grapnel rather than NEDD1, that bind to GCP5 and GCP6. However, two of the extended C-terminal tails of the NEDD1 tetramer outside the grapnel, directly interact with GCP2 (position 1), GCP3 (position 2) and GCP6 (amino acids 223–240). The remaining pair of extended C-terminal NEDD1 tails does not associate with GCPs.

Tetramerization of NEDD1 is essential for γ-TuRC binding, as mutations in its C-terminal alpha-helix that disrupt bundle formation abolish this interaction [25, 61]. The binding of the C-terminal NEDD1 tetramer to four MZT1-N-GCP3 sites located at distinct positions within the γ-TuRC likely ensures that only the fully assembled ring complex can interact with NEDD1 and be recruited to the centrosome.

Besides CEP192/NEDD1, the PCM protein CDK5RAP2 with its CM1 motif has also been implicated in γ-TuRC binding. In cryo-electron microscopy studies, Xu et al. [62] and Gao et al. [25] observed that binding of CDK5RAP2 and NEDD1 to γ-TuRC was not mutually exclusive, contrary to earlier suggestions based on pull-down experiments [63]. Interestingly, depletion of CDK5RAP2 has only a mild effect on the interphase PCM localization of γ-TuRC at least in RPE1 cells [25, 64], while depletion of CEP192 strongly disrupts γ-TuRC localization within the PCM [26]. During interphase CEP192/NEDD1 likely recruits the γ-TuRC to the PCM while this is not the major function of CDK5RAP2. Instead, mounting evidence indicates that CDK5RAP2 activates the γ-TuRC by inducing structural changes as outlined in detail below [25, 62, 65]. Surprisingly, in RPE1 cells, CDK5RAP2 is not essential for viability [64], indicating the existence of γ-TuRC activation mechanisms that operate independently of CDK5RAP2. Kinases associated with CEP192 may regulate the structural conformation of the γ-TuRC, thereby promoting its activation. Interestingly, although CDK5RAP2 is normally dispensable for mitotic spindle formation, it becomes essential for spindle assembly via the acentrosomal pathway when centrioles are absent, as in cells treated with the Plk4 inhibitor centrinone [66].

How does CDK5RAP2 activate the γ-TuRC for microtubule nucleation on a molecular level? CDK5RAP2 contains a conserved CM1 domain [67]. An isolated fragment encompassing this motif (CDK5RAP2^44–93^​) was previously used to affinity-purify human γ-TuRC [14], indicating a direct interaction between CDK5RAP2 and γ-TuRC. Moreover, overexpression of this fragment was shown to promote microtubule nucleation [67], suggesting that the CM1 of CDK5RAP2 acts as an activator of the γ-TuRC. Work using porcine (*S. crofa*) brain lysate [62] or recombinant human γ-TuRC [65] incubated with a 20-fold molar excess of recombinant CM1 revealed that the CM1 motif can bind to multiple positions within the γ-TuRC [62]. These sites are marked by the N-GCP2–CM1 complex with MZT2, a paralogue of MZT1, which, however, is not part of the γ-TuRC core. In addition to position 13, only the following combinations of positions were occupied: position 7 alone, positions 5 and 7; or positions 3, 5, and 7. No other configurations were observed. At position 1, no clear density for MZT2 was detected, which may indicate a lower affinity or the flexible binding of MZT2 for this particular N-GCP2. In contrast, the recombinant human γ-TuRC that was incubated with dimeric CM1 (EB1 dimerization domain) contained the CM1 at all GCP2 positions (1, 3, 5, 7 and 13) [65]. The discrepancy between the two CM1 studies may be due to EB1-induced dimerization of CM1 or to differences in the γ-TuRC sources (*S. crofa* brain versus recombinant human γ-TuRC) [62, 65].

Interestingly, even without enforced dimerization, CM1 was observed as a dimer bound to GCP2 and spanning the GCP2–GCP3 interface [68]. This behavior was also seen for CM1 from the pathogenic yeast *C. albicans* and the budding yeast *S. cerevisiae*, indicating that it is a conserved feature [69]. Notably, in these yeasts, CM1 binding occurs independently of MZT2, which is not encoded in these genomes [70].

As outlined above, depending on the conditions, the isolated CM1 can bind to different GCP2 positions within the γ-TuRC. This raises the question of how full-length CDK5RAP2 binds to the γ-TuRC. Gao et al. [25] addressed this by analyzing CDK5RAP2 decoration of γ-TuRCs in purified centrosomes, revealing that CDK5RAP2 interacts via its CM1 motif with MZT2-N-GCP2 modules either at position 13 or positions 1, 3, 5, and 7 (Fig. 1e). These data indicate that the CM1 fragment does not fully recapitulate the behavior of full-length CDK5RAP2 and, therefore, results obtained with CM1 in vitro may not accurately reflect the in-situ situation.

Importantly, all three studies demonstrated that CM1/CDK5RAP2 binding has the potential to influence the conformation of the γ-TuRC [25, 62, 65]. CDK5RAP2 binding exclusively at position 13 was associated with the more inactive, outward conformation that deviates from microtubule symmetry (Fig. 1d and e). However, CDK5RAP2 binding to the MZT2-N-GCP2 modules 1, 3, 5 and 7 of the γ-TuRC reorients γ-tubulin molecules at positions 1–9 into a tubulin-like geometry. In addition, γ-tubulin molecules at positions 13 and 14 shifted inward, reducing their Euclidean distance from microtubule-compatible symmetry. This structural impact by CDK5RAP2 binding that is also seen by the isolated CM1 explains the observed activation of γ-TuRC–mediated microtubule nucleation upon CM1 overexpression in human cells [67]. In summary, the CM1 motif of CDK5RAP2 binds to MZT2-N–GCP2 modules within the vertebrate γ-TuRC, modulating its geometry towards microtubule symmetry, thereby enhancing γ-TuRC activity.

The question remains how γ-TuRC achieves microtubule-like symmetry after only partial closure induced by CDK5RAP2 binding. Recent studies on microtubule minus ends suggest that γ-TuRC may not always attain full symmetry following nucleation [71]. Alternatively, additional regulators, such as the Nucleoside-Diphosphate Kinase NME7, which associates with γ-TuRC in an as-yet unclear manner and promotes its activation, could influence its structure [72]. Another possibility is that forces generated during microtubule formation affect γ-TuRC conformation, as suggested by in vitro studies using a GTPase-defective tubulin mutant [73].

Actin binds to the γ-TuRC through MZT1-N-GCP6 and γ-tubulin at position 2 [12–15]. When the N-terminus of GCP6 is removed, actin no longer binds to the γ-TuRC (ΔN-GCP6). Despite this deficiency, the γ-TuRC^ΔN−GCP6^ can still assemble and retains microtubule nucleation activity in vitro. Interestingly, however, in the absence of actin binding, the two γ-tubulin molecules at positions 1 and 2 are displaced outward within the complex. These structural defects in γ-TuRC^ΔN−GCP6^ affect its cellular function, although the cells remain viable. Cells expressing *ΔN-GCP6* exhibit defects in microtubule nucleation and mitotic chromosome segregation [74].

Previous studies suggested that actin can be released from the γ-TuRC during microtubule nucleation [73] although this was not uniformly observed [75]. Consistent with the release of actin, Serna et al. [65] reported that activation of γ-TuRC by CM1 dimers decreases actin occupancy at position 2 in approximately 30% of γ-TuRC complexes. However, both subpopulations, regardless of luminal actin presence, display CM1 dimer density at all five binding sites, indicating that CM1 occupancy does not depend on actin, although bound CM1 dimers can trigger actin release in some complexes. Further studies are needed to elucidate the function of actin in the γ-TuRC. An equally important question is whether actin binding to the γ-TuRC is evolutionarily conserved, as it is currently unknown whether actin is a component of the GCP2-6 based γ-TuRC in organisms outside of vertebrates.

#### γ-TuRC and augmin in the lumen of centrioles

Karsenti and colleagues, using immuno-electron microscopy, first observed that γ-tubulin is not restricted to the PCM but is also present within the centriole lumen [76]. The localization of γ-tubulin within the centriole lumen was further investigated by Lüders and colleagues [26]. They demonstrated that the γ-TuRC is anchored inside centrioles through the combined action of NEDD1, the augmin complex, and the inner scaffold protein [50]. In contrast, CEP192, the protein responsible for recruiting γ-TuRC to the PCM, does not contribute to its luminal localization. Disruption of γ-TuRC localization in the centriole lumen, achieved by depleting the augmin subunit HAUS6, decreased centriole number in cells arrested for 18 h in prometaphase by treatment with the Eg5 motor protein inhibitor STLC. This decrease in centriole number was interpreted as centriole disintegration over time. From these observations, the authors concluded that γ-TuRC and augmin within centrioles provide a stabilizing function similar to that of the inner scaffold proteins [77, 78].

Recently, it was confirmed that augmin, NEDD1, and POC5 mediate the recruitment of the γ-TuRC to the centriole lumen [25]. In addition, cellular cryo-electron tomography of centrosomes in vitreous sections of human HCT116 and HeLa cells, combined with subtomogram analysis, was used to map the distribution of γ-TuRCs within the native centrosome at single-molecule precision. Interestingly, this analysis revealed a defined spatial organization of γ-TuRCs inside each centriole (Fig. 2c). Specifically, γ-TuRC molecules were found to localize in the centriole lumen, approximately 75 nm away from the B-microtubule wall. These γ-TuRCs were densely packed, with a spacing of ~ 25 nm. Unlike the PCM-localized γ-TuRCs, the lumenal γ-TuRCs displayed a defined orientation: the GCP4–5–4–6 interface was consistently oriented toward the microtubule wall (Fig. 2c). This indicates the presence of a dedicated tethering mechanism that anchors γ-TuRCs within centrioles and restricts their flexibility.

What are the mechanisms by which POC5, augmin, and NEDD1 mediate the tethering of the γ-TuRC to the centriole lumen? The positioning of γ-TuRC within centrioles is mediated through interactions between the TII subcomplex of augmin (HAUS2, HAUS6, HAUS7, and HAUS8) and the inner scaffold protein POC5 [25] (Fig. 2c). AlphaFold predicts that the HAUS2 and HAUS7 subunits bind POC5 within amino acids 224–235. Consistently, mutational analysis of POC5 (S224E, R227A, K228A, V231A, S234K, L235A, termed POC5^mut^) disrupted the predicted augmin-binding interface, experimentally confirming its role in mediating augmin interactions [25].

The elongated augmin complex spans the central part of the centriole lumen, with its TII subcomplex anchored near the microtubule wall through binding to POC5 [25] (Fig. 2c). Whether the microtubule-binding elements of augmin, namely the N-terminus of HAUS8 and the CH domain of HAUS6 [79], contribute to anchoring augmin to the centriole microtubule wall remains unclear. AlphaFold predictions and biochemical evidence further suggest that the NEDD1 grapnel interacts with both the γ-TuRC and the augmin TIII subcomplex. Consistent with these interactions and topological predications, the TIII subunit HAUS1 is positioned in the centriole lumen close to the γ-TuRC as indicated by MINFLUX Nanoscopy [25].

POC5 forms a tetrameric complex and each POC5 subunit binds several centrin molecules [25, 50], which is an evolutionarily conserved MTOC-associated protein [80]. The POC5 tetramer has the potential to crosslink with multiple binding partners, namely POC1A, POC1B, CCDC15, and WDR90, which together constitute the centriole inner scaffold that also binds to the centriole microtubule wall [77, 78, 81]. Thus, the POC5 network, along the inner surface of the centriole microtubule wall, connects to the NEDD1–γ-TuRC in the lumen through the elongated augmin complex (Fig. 2c).

Lüders and colleagues, by depleting subunits of the augmin complex—thereby disrupting its overall function not only within centrioles but also in mitotic spindle assembly—concluded that augmin–γ-TuRC is essential for both centriole integrity and centriole length [26]. To investigate this function more specifically, Gao et al. [25] employed the *POC5*^*mut*^ mutant, which is deficient in augmin recruitment to the centriole lumen but still assembles a functional centriole inner scaffold, in combination with HAUS1-SNAP localization to detect the augmin complex. This analysis revealed three key findings. First, in wildtype *POC5* cells, the augmin and γ-TuRC are released from the centriole lumen at the onset of mitosis through a mechanism dependent on polo-like kinase PLK1. Second, the absence of luminal augmin–γ-TuRC accumulation in *POC5*^*mut*^ cells did cause mitotic defects. Third, *POC5*^*mut*^ cells exhibited reduced cellular levels of augmin and γ-TuRC. Cycloheximide chase experiments assessing protein stability further revealed decreased stability of γ-tubulin and the HAUS6 subunit in *POC5*^*mut*^ cells compared to wild type *POC5* cells. Together, these results support the inner-centriole protection model in which luminal augmin–γ-TuRC complexes are protected from degradation during interphase and released from centrioles at the onset of mitosis to fulfill a mitosis-specific role.

Although the inner-centriole protection model is appealing, several uncertainties remain that need to be addressed in future experiments. Fluorescence recovery after photobleaching (FRAP) experiments indicate that augmin and γ-TuRC are relatively stable bound to the centriole lumen in interphase cells. However, because FRAP measurements are performed over a relatively short time frame, much shorter than an entire cell cycle, it cannot be excluded that slow exchange of augmin and γ-TuRC occurs within the centriole pool during interphase. An alternative possibility to the inner-centriole protection model is that cytoplasmic POC5 contributes to the stabilization of augmin and γ-TuRC, although a substantial cytoplasmic pool of inner scaffold proteins has not yet been described. An important unresolved question concerns the relative size of the γ-TuRC pool inside centrioles compared to the total cellular pool [51, 52]. The same applies to augmin.

How the release of augmin and γ-TuRC from centrioles at the onset of mitosis contributes to mitotic spindle formation remains an open question. However, considering the regulation of cytoplasmic augmin by RanGTP and importin [82, 83], as well as its release from centrioles, we propose a dual-spatial activation for augmin. Cytoplasmic augmin, associated with importin, is likely kept inactive until nuclear envelope breakdown during prometaphase. At that point, RanGTP releases importin from augmin, enabling augmin to bind to microtubules and interact with NEDD1–γ-TuRC promoting microtubule branching, particularly near chromatin enriched in the Ran guanine nucleotide exchange factor (RanGEF) RCC1 that activates the GTPase Ran and releases augmin from importin [84, 85]. Even if centriolar augmin and γ-TuRC represent only a small fraction of the total cellular pool, centriole released augmin and γ-TuRC could locally promote the formation of branched microtubules near spindle poles, thereby contributing to spindle assembly. These rapid and localized mechanisms of γ-TuRC and augmin activation are well suited to the swift and three-dimensional assembly of the mitotic spindle and the accompanying burst of microtubule nucleation at the onset of mitosis, which is completed within minutes.

#### The procentriolar microtubule assembly and the role of γ-TuRC

The nine-fold symmetric cartwheel stack assembled on the surface of the mother centriole serves as an organized scaffold that initiates the assembly of the centriolar triplet microtubules [38]. Though the cartwheel framework and the triplet microtubule architecture appear to co-exist for a significant time during the procentriole assembly, the molecular connections between the two have begun to be uncovered only recently. Cryo-electron tomography of centrioles isolated from human cells revealed asymmetric cone like structures at the bottom of the A-microtubules during the early procentriole stage cells, which disappear shortly after initiation of A-microtubule assembly. Given the cap-like appearance of the structures at the nucleation site of the A-microtubules, the authors speculated them as γ-TuRCs. This work also revealed the absence of such γ-TuRC-like structures at the bottom of either B- or C-microtubules, implying the γ-TuRCs are not required for their formation (Fig. 4ai) [86]. Though regulations of B- and C-microtubule assembly mechanisms are less known, earlier work in unicellular eukaryotes including *Chlamydomonas* and *Paramecium* indicated involvement of δ- and ε-tubulin in B- and C-microtubule stability, since their mutations result in centrioles only with singlet microtubules [87–89]. Recently, de-novo centriole formation in δ- and ε-tubulin knockout RPE-1 cells also showed reduction in the outer diameter of the centrioles, supporting the earlier findings in lower organisms. These mutant cells also have been shown to generate multiple centrioles de-novo, but they are less stable and are incapable of duplication (Fig. 4aii). Interestingly, these δ- and ε-tubulin knockout centrioles, appear to recruit SAS-6, but they lack the inner scaffold protein POC5 and γ-tubulin, implicating a possible role of doublet or triplet formation in the recruitment of these proteins (Fig. 4bii) [90].

Regarding the nucleation of A-microtubules, the precise location of the γ-TuRC cap and how γ-TuRC is activated at this site remain largely unknown. However, a recent study supported the role of cartwheel protein SAS-6 in this process. Specifically, the C-terminal regions of SAS-6, which project outwards towards the centriolar wall, play an essential role in stabilizing γ-TuRC at the procentriole initiation site. Supportively, removal of the C-terminal ~ 200 amino acids of SAS-6 led to the loss of procentriolar microtubule formation [91]. It remains unclear, whether SAS-6 C-terminal extensions throughout the whole cartwheel length are involved in γ-TuRC stabilization. A more recent study showed that procentriole starts assembling at the very early stage of cartwheel assembly. However, the cartwheel stack also appears to grow towards the mother centriole wall, resulting in the translocation of the procentriole microtubule structure away from the mother centriole surface (Fig. 4bi) [91, 92].

The mechanisms of how the γ-TuRCs are organized, recruited and translocated along the procentriolar microtubules awaits further studies. It is also important to understand whether any other cartwheel and centriolar proteins are involved in γ-TuRC regulation in cooperation with SAS-6. A prominent regulator could be CEP135, which binds to both SAS-6 and centriolar microtubules and organizes onto the pinheads connecting the edges of the cartwheel structure [45, 93, 94]. Earlier studies showed that the abrogation of CEP135 disrupts centriolar microtubule triplet structure. Moreover, CEP135 also interacts with CPAP, a factor that promotes centriolar microtubule growth [94, 95]. CPAP also appears to have a more direct connection with the cartwheel and the γ-TuRC, as it binds to STIL near the STAN domain, where also SAS-6 binds. Immuno-affinity analyses of both centrosomal and cytosolic human cell extracts also showed CPAP association with γ-tubulin [96]. However, whether these accessory proteins regulate γ-TuRCs at the cartwheel or A-microtubule nucleation level remains to be determined.

After centriole microtubules assemble near the wall of the mother centriole around the SAS-6 cartwheel, they elongate outward, while the emerging centriole lumen becomes progressively filled with proteins. Ultra-expansion microscopy and cryo-electron tomography in human cells have shown the localization of γ-tubulin, the core γ-TuRC protein GCP4 and other γ-TuRC proteins including NEDD1 in different regions of the mother and the growing daughter centrioles. Unlike the older centriole, the core γ-TuRC protein GCP4 and NEDD1 remain mostly confined to the outer microtubule wall during the early growth of the daughter centriole [26]. Once it reaches an intermediate length, the γ-TuRC, in addition to its outer wall association, an additional pool appears to be present in the luminal region. NEDD1 also behaves the same. HAUS6/augmin appears to be present majorly in the inner wall of mature centriole and POC5 also seems to be present in this region (Fig. 4bi). It is possible that the newly assembled POC5 facilitates HAUS6/augmin recruitment to the inner wall, which follows γ-TuRC/NEDD1 recruitment. NEDD1 may favor bridging between HAUS6/augmin and γ-TuRC [25, 26] (Fig. 2c). It is also to note that the structure of the proximal centriolar region is minimally affected in the *HAUS6* knock-out cells, suggesting its involvement as stabilizer to be unlikely during procentriole initiation.

An additional interesting link of the NEDD1 with centriolar cartwheel has been reported showing that its N-terminal WD40 domain associates with SAS-6, in a Plk4 phosphorylation-dependent manner. Interestingly, NEDD1 in its phosphorylated state, interacting with SAS-6, exhibits no association with the γ-TuRC with its C-terminus in immunoprecipitation experiments [59, 97]. Cells expressing a phospho-deficient NEDD1 mutant incapable of binding to SAS-6, however, regain the NEDD1-γ-TuRC interaction [59]. These data are in support of a broader role of NEDD1 in stabilizing the cartwheel on one side and presumably, recruiting the γ-TuRCs to the cartwheel peripheral region to nucleate the A-microtubules on the other side. However, structural arrangements involved in mediating these processes and their temporal control remain to be investigated. It is also interesting to note that unlike NEDD1, which is present both in the centriolar lumen and the procentriole assembly site on the mother centriole, Plk4 is only enriched at the proximal mother centriole toroidal surface, but not in the lumen [41, 43]. This brings a speculation that two distinct NEDD1 pools may function separately in cartwheel stabilization and the γ-TuRC recruitment.

### γ-TuRC at the sub-distal appendages

The older parental centriole organizes extra proteinaceous appendage-like structures towards its distal end, namely the distal (DAs) and sub-distal appendages (SDAs) [98]. The distal appendages, which appear much more organized and widely extended structures projecting outward from the centriolar microtubule surface, make the older parental centriole unique in recruiting numerous other proteins to facilitate assembly of other centriole-derived organelles such as cilia. During ciliogenesis, distal appendages facilitate docking of the mother centriole (now called the basal body) to the cell membrane, a critical step for cilia formation, and serve as a transitional scaffold between the mother centriole and the axoneme, the core microtubule-based structure of eukaryotic cilia organized by the microtubules of the basal body. Distal appendages primarily organize into a nine-fold symmetric, conical-shaped architecture, consisting of nine blades crowned with proteins such as SCLT1 and CEP83, which further mediates the recruitment of CEP164. Each of these proteins exhibits a distinct localization to the blades, with CEP164 at the distal tip of the blades, and cumulatively appear to link the microtubule doublets of the mother centriole [99–101]. These blades are intervened by the presence of ciliary proteins FBF1 and IFT88, which mark the distal appendage matrix (DAM). The distal appendage proteins, CEP164 and SCLT1 have been shown to be crucial for cilia initiation, as knockout of these genes fails to dissociate CP110 from the mother centriole and abrogates cilia formation [101, 102]. The Tau tubulin kinase 2 complexes with CEP164 and phosphorylates it, in order to activate the CEP164 for transitioning the cell towards ciliogenesis [103]. However, the loss of DAM protein, FBF1, can still lead to cilia initiation, but transport of ciliary membrane proteins gets adversely affected, as has been observed in *FBF1* knockout cells [101].

Though there is no clear evidence yet if the γ-TuRC proteins could have a role in organization of distal appendages, recent studies supported their presence at the SDA region. Though the proteins like Ninein and Cenexin were identified to be involved in microtubule assembly around the mother centriole via their association with the SDA structures [104, 105], a better molecular link of the SDAs with microtubule nucleation became clearer with the recent high-resolution based analysis of these structures showing the presence of γ-tubulin and some of its associated proteins in this region [106]. Cytoskeleton-associated protein 5 (CKAP5/ch-TOG/XMAP215), which was originally identified as a microtubule polymerase [107, 108], has been shown to recruit and activate the γ-TuRC at the SDAs, in addition to its association with the PCM and the microtubules emanating from the centrosome. Specifically, analyses of human interphase centrioles showed that ch-TOG co-localizes with Ninein and a low density of γ-tubulin. However, immunoprecipitation analysis failed to detect any interaction of ch-TOG with γ-tubulin in this condition [106] and the same has been observed in another study [109]. However, pull-down assay by biotinylated GCP3 could detect its interaction with cellular ch-TOG along with γ-tubulin, suggesting the interactions between the endogenous proteins may be very transient [106]. In line with these results, studies using purified proteins from *Xenopus laevis* did show a detectable interaction between γ-tubulin and XMAP215 [110]. Analyses of ch-TOG knockdown cells further demonstrated loss of γ-tubulin from the SDA, but not in the centriolar lumen, further supporting the specific role of the SDA localized pool in microtubule aster formation [106]. ch-TOG/XMAP215 also binds to transforming acidic coiled coil protein 3 (TACC3) [109, 111, 112], which is also essential for centrosomal microtubule assembly, and centrosomal recruitment of both proteins requires Aurora A kinase activity [113–115]. However, it remains unclear whether γ-TuRC organization at the SDAs also involves the chTOG-TACC3 axis.

#### γ-TuRC and its associated centriolar biogenesis proteins in diseases

Duplication frequency of centrioles requires a tight control during the cell cycle as both the length and number aberrations of centrioles cause defects in spindle polarity, chromosomal mis-segregation and cilia dysfunction [116, 117]. Consequently, in many cases, centrosome/centriole amplification has been considered as a driving mechanism leading to numerous cancers including solid tumors, microcephaly and certain cilia related disorders, commonly termed as ciliopathies [118]. Apart from the cell biological aspects of γ-tubulin and its complexes at the molecular level, there has been an increasing attempt to delineate their links to diseases using whole organisms and clinical samples. Studies in zebrafish elucidated the essentiality of GCP3 for retinal development. Specifically, GCP3 knockout causes developmental defects in the peripheral region of the retina, where proliferating ciliary marginal zone cells are abundant. This happens to be mediated via mitotic arrest and apoptosis in that region [119]. In case of human patients, a homozygous mutation of conserved Glu311 to Lys in GCP2 in twins from consanguineous parents, have been found to cause developmental defects in the brain leading to specific dysmorphic features [120]. Analysis of those patients’ fibroblast cells revealed significant loss of centrosomal recruitment of γ-tubulin, HAUS6 and NEDD1 specifically in mitotic cells. Further proteomics analysis of the patient cells also revealed differential expression patterns of specific tubulin isotypes, whose functions are implicated in brain development. *GCP2* mutations are also linked to diseases associated with metabolic dysfunctions in neuronal cells and associated developmental defects in the brain. Specifically, the expression level of PHGDH, the enzyme essential for L-serine production during brain development, is down-regulated in the patient derived *GCP2* Glu311Lys mutant cells [120–122]. Analysis of human γ-TuRC cryo-EM structure further implicated a destabilizing effect on GCP2-GCP3 interaction and the γ-TuRC stability as a result of this mutation [120]. Whole exome sequencing of two families and Sanger sequencing of twelve families with members having autosomal-recessive microcephaly and chorioretinopathy, revealed compound heterozygous mutations in *GCP4*, which included exon skipping induction, frameshift mutation and deletion mutation. Specifically, analysis of patient derived fibroblast cells carrying *GCP4* mutation associated with exon 16 skipping, showed impairment of γ-TuRC assembly with microtubule nucleation defects and those cells also exhibited several nuclear abnormalities reminiscent of aneuploidy [123]. Furthermore, biallelic variants of both *GCP4* and *GCP6* have also been linked to microcephaly and chorioretinopathy [124]. Additionally, Phe727Cys mutation in *GCP5* also has been shown to cause developmental delay and is attributed to primary microcephaly [125]. In addition, the autosomal dominant mutation in human γ-tubulin is associated with lissencephaly and microcephaly [126].

Apart from these brain related disorders, mutation/deletion of γ-TuRC proteins are also linked to cancer. Specifically, both missense mutation and in-frame deletion of *GCP6* have been found in several patients having relapsed pancreatic cancer [127].

Mis-regulation of microtubule nucleation as a result of loss of function of its cooperating factor could also have implications in diseases. Supportively, knockout of *HAUS6* in mice has been shown to cause developmental defects in the brain, attributing to mitotic delay and p53 mediated apoptosis [128]. Though it remains unclear whether the disease manifestation by γ-TuRC proteins is associated with their PCM or the centriolar pools, mutations of core centriole proteins have also been implicated in diseases. For example, mutation/deletion of KEN box motif involved in STIL degradation by ubiquitin ligase APC/C has been found to be associated with primary microcephaly [129–131]. Overexpression of *STIL* and associated centrosome amplification has been observed in non-small-cell lung cancer patients [132]. An Ile62Thr mutation in the cartwheel protein SAS-6 was found to be homozygously present in individuals affected with primary microcephaly [133]. Collectively, these results demonstrate that the human microtubule system is critical for development, and its dysfunction may contribute to cancer development (Table 1).

**Table 1:** Mutations and diseases linked to γ-TuRC and its associated proteins

Gene name	Associated diseases	Mutations/KO	Cellular phenotypes
*Tubgcp3*	Retina developmental disorder	KO [119]	Mitotic arrest/apoptosis
*Tubgcp2*	Brain developmental disorder Brain metabolic dysfunction	Glu311Lys [120]	Loss of γ-tubulin, HAUS6 and NEDD1 from centrosome Destabilized γ-TuRC
*Tubgcp4*	Congenital Microcephaly	Gly194Trp Tyr100Ile [123]	Impaired γ-TuRC assembly Microtubule nucleation defect
*Tubgcp5*	Primary microcephaly	Phe727Cys [125]	N.A.*
*Tubgcp6*	Pancreatic ductal adenocarcinoma and multiple other cancers	Val1472Leu Leu1491X Val158Leu Glu1429Gln Glu115Gln Trp1448Cys Leu1419Met Leu105Met Thr1438X Total of 376 somatic mutations: https://portal.gdc.cancer.gov/genes/ENSG00000128159	N.A
*Tubg1*	Lissencephaly (Cortical dysplasia) and Microcephaly	Glu177Gly [126]	N.A
*HAUS6*	Brain developmental defect	KO [128]	Mitotic delay p53 mediated apoptosis
*SAS-6*	Primary microcephaly	Ile62Thr [133]	N.A
*STIL*	Primary microcephaly	ΔKEN box motif Val1219X [129]	Centriole Amplification

*N.A., Not assessed

## Data Availability

Not applicable.
